# Translation, cross-cultural adaptation and validation of the Chinese version of the IBD-Control questionnaire: A patient-reported outcome measure in inflammatory bowel disease

**DOI:** 10.1371/journal.pone.0311529

**Published:** 2024-12-12

**Authors:** Bingmei Guo, Haihong Li, Qing Cui, Jie Li, Yanbo Yu, Zhen Li, Junwen Wang

**Affiliations:** 1 Department of Gastroenterology, Qilu Hospital of Shandong University, Jinan, People’s Republic of China; 2 Nursing Theory & Practice Innovation Research Center of Shandong University, Jinan, People’s Republic of China; 3 Department of Toxicology and Occupational Diseases, Qilu Hospital of Shandong University, Jinan, People’s Republic of China; King Abdulaziz University Faculty of Medicine, SAUDI ARABIA

## Abstract

**Background:**

A demand exists for user-friendly patient-reported outcome measures for patients with inflammatory bowel disease (IBD). The IBD-Control Questionnaire has been recently developed to assess overall disease control from the patient’s view but has not been available in China.

**Methods:**

Translation and cultural adaption of the IBD-Control into Chinese was conducted through four steps (forward translation, backward translation, expert panel, and pilot testing). Afterwards, a prospective validation study was conducted from February 2022 to February 2023. The translated IBD-Control, Short Health Scale, EQ-5D-5L, and disease activity measurements using either the Physician Global Assessment and Simple Clinical Colitis Activity Index or the Crohn’s Disease Activity Index were used. Acceptability, test-retest reliability, internal consistency, content validity, convergent validity, structural validity, discriminant ability, and receiver operating characteristic curves were analyzed.

**Results:**

Questionnaires were completed by 150 participants with IBD (31 with Crohn’s disease [CD] and 119 with ulcerative colitis [UC]). The Cronbach’s alpha coefficient was 0.823 for the IBD-Control-8 scale. The correlations between individual item and total score varied from 0.485 to 0.892 among CD patients and from 0.588 to 0.712 among UC patients. The S-CVI/Ave was 0.98. Convergent validity analyses exhibited moderate to strong correlations between other measurements and IBD-Control-8-subscore (0.555–0.675) or IBD-Control VAS (0.503–0.671). Test-retest analysis showed that the mean scores were 75.23±17.33 versus 72.10±14.99 (*r* = 0.894, *p<*0.01) for VAS scores and 12.75±3.49versus 12.80±3.29 for IBD-Control-8 subscore (*r* = 0.963, *p<*0.01), respectively. The IBD-Control-8-subscore and IBD-Control-VAS exhibited significant discriminative capability among the PGA categories (ANOVA, *p* < .001). The ROC analysis revealed an optimal cut-off point for the IBD-Control-8 subscore of 14 points (sensitivity: 70.9%, specificity 83.5%), versus a cut-off of 70 on the IBD-Control VAS score (sensitivity: 84.4%, specificity 69.3%).

**Conclusion:**

The Chinese IBD-Control proves to be a disease-specific, reliable, and valid tool for revealing overall disease control from the patient’s viewpoint. Both healthcare professionals and patients may find it to be a useful patient-reported outcome measurement for triaging IBD patients in China or complementing routine care.

## Introduction

Inflammatory bowel disease (IBD) is a chronic disorder characterized by inflammation of the gastrointestinal tract, encompassing two forms: Crohn’s disease (CD) and ulcerative colitis (UC). IBD was initially found in Western countries during the industrial revolution [1]. With the increase in industrialization and Westernization of lifestyles in China, there has been a rapid and significant increase in the incidence rate of IBD in recent years [2,3]. Goals in the treatment of IBD are normally determined by clinical, endoscopic, biological, and histological endpoints provided by medical staff [4,5]. However, evidence shows a difference between the assessments of medical staff and those of patients with IBD [6]. Patient-reported outcomes (PRO) refer to the health status and treatment results reported directly by the patient, without interpretation by a clinician or anyone else [7]. In IBD, the incorporation of PROs alongside clinical scores used by clinical physicians can enhance predictive capacity for endoscopic remission, assessment of treatment efficacy, and monitoring of adverse reactions [8,9].

Despite the wide variety of IBD-specific, patient-reported outcome measurements (PROMs) developed over the past two decades [10], none have been widely used in daily clinical practice. One possible reason is that most PROMS are lengthy multi-domain questionnaires and their use imposes a significant burden on patients. Another reason is that all these instruments focus mainly on quality of life (QoL), overall well-being, vitality, physical discomfort, as well as the effects of the disease on physical, social, and emotional functioning; however, none of them accurately capture the patients’ perspective on disease control, which is an important goal of therapy in IBD.

The IBD-Control questionnaire was the first validated, user-friendly tool to capture a patient’s view on disease control by examining various aspects of symptoms, treatment effectiveness, and overall QoL [11]. This questionnaire is recommended by the International Consortium for Health Outcomes Measurement [12]. Unlike most PROMS for IBD, IBD-Control was developed in collaboration with patients and can directly reflect patients’ subjective perceptions of disease control. The IBD-Control questionnaire holds potential utility in clinical settings, offering a concise assessment tool capable of discerning patients in a state of remission, which bears significant clinical ramifications, potentially sparing patients with well-managed disease from requiring in-person consultations with clinicians. In addition to the ability to detect patients with ‘quiescent disease’, the IBD-Control may stimulate patient-empowerment by revealing the topics that patients want to discuss during their next outpatient visit. The questionnaire comprises four sections consisting of 13 categorical items along with a visual analogue scale (VAS). Disease control perception is assessed using an eight-item scale comprising the first and third sections, along with a VAS. This eight-item scale has a total score range of 0 to 16 and the VAS ranges from 0 to 100 with higher scores indicating a higher perception of disease control. The IBD-Control questionnaire has been translated, validated, and applied in several countries [13–15].

In China, the healthcare resources are strained, and the economic burden on IBD patients is relatively high [16,17]. Many IBD patients in China are unable to undergo regular follow-up examinations due to the limited accessibility and affordability of healthcare services. In this context, the availability of validated IBD-Control questionnaire could be particularly valuable. It could empower patients to self-monitor their condition, reduce the need for frequent in-person consultations, and optimize the utilization of limited healthcare resources. Furthermore, the validated Chinese version of the IBD-Control questionnaire would enable Chinese IBD patients to actively participate in the management of their disease, potentially improving treatment adherence and outcomes [18]. However, there is currently no validated IBD-Control questionnaire available for use in patients with IBD in China.

This study aimed to (1) provide a Chinese translation and cultural adaption of the IBD-Control questionnaire, (2) validate the IBD-Control questionnaire in a Chinese area, and (3) further detect the optimal cut-off scores of the IBD-Control to identify patients with a state of ‘quiescent’ in Chinese population.

## Materials and methods

### Design

The original IBD-Control questionnaire was translated into Chinese, and the content validity was assessed by administering several cross-sectional questionnaires to healthcare professionals (HCPs). Thereafter, a prospective monocentric study was undertaken at a tertiary hospital’s department of gastroenterology in China. Validity, reliability, discriminant ability and optimal cut-off scores of the translated version was evaluated.

### Process of the translation

Authorization to translate and use the instrument was obtained from the original author. The translation and adaptation process was based on the COSMIN Study Design checklist for patient-reported outcome measurement instruments [19]. The translation process consisted of four steps.

Step 1: Forward Translation. Independently, two nurses (one with a master’s degree in nursing and attained Level 6 English proficiency, while the other with a PhD in nursing) who were native Chinese speakers and proficient in English conducted the translation of the original version into Chinese. The translation coordinator consolidated and revised the two Chinese translations to create the initial Chinese version of the IBD-Control questionnaire.

Step 2: Backward translation: Two translators (one was knowledgeable about patient-reported outcomes and the other was excellent regarding the cultural and linguistic subtleties of the English language) independently back-translated the first draft of the mainland Chinese version into English. Both translators were blinded to the original IBD-Control questionnaire. Multidisciplinary experts conducted a comparative analysis of the original questionnaire with the two back-translated versions to evaluate the consistency of meaning, terminology accuracy and clarity of expressions. After language adaptation, the second version of the IBD-Control questionnaire was formulated.

Step 3: Expert panel. In this study, six experts (physicians, nurse scientists, registered nurses, and professors) with expertise in IBD care or patient-reported outcomes were invited to present their opinions on the comprehensibility of language and content. Subsequently, each expert was asked to evaluate the translations on a four-point Likert scale with the following options: 1 indicating not relevant, 2 indicating unable to assess relevance, 3 indicating relevant but requires minor alterations, and 4 indicating very relevant and succinct. The average content validity index (S-CVI/Ave) was computed at the scale level, with a threshold of ≥0.90 deemed as an acceptable criterion. After integrating expert opinions, the third version of the IBD-Control questionnaire was developed.

### Psychometric validation

#### Population and statistical sample

This validation study was conducted in Jinan, Shandong Province, China, from February 2022 to February 2023. Hospitalized patients with IBD in the department of gastroenterology of Qilu Hospital were recruited for the study.

Inclusion criteria were: 1) inpatients diagnosed with IBD based on the Consensus on the Diagnosis and Treatment of Inflammatory Bowel Disease in China [20], 2) patients aged between 16 and 80 years, and 3) willingness to participate in the study.

Exclusion criteria were: 1) patients with significant complications such as heart failure, respiratory failure, or cancer, 2) pregnant women, and 3) patients with severe cognitive impairment that hindered their ability to comprehend and respond to the questionnaire.

The sample size for our study was determined based on the recommended rule of having a minimum of 10 respondents per scale item [21]. Given that the Chinese IBD-Control questionnaire comprises 14 items, we aimed to recruit at least 140 participants to ensure adequate statistical power and reliability of the psychometric analyses.

#### Measures

Baseline characteristics, including age, gender, marital status, diagnosis, duration (years), medication, perianal disease and history of operation, were retrieved from medical records. The disease activity was measured using the Harvey-Bradshaw index (HBI) for CD [22] or the Short Clinical Colitis Activity Index (SCCAI) [23] for UC. The Physician Global Assessment (PGA) was also utilized to record disease activity, such as mild, moderate, and severe activity, as well as remission. Four specially trained healthcare professionals who were blinded to the results of the questionnaires completed by the patients, independently completed the HBI or SCCAI and PGA within 24h after the patients finished the questionnaire.

Patients were asked to complete the Chinese version of the IBD-Control, a short health scale (for evaluating the QoL among patients with IBD), and the EQ-5D-5L (a visual analog scale [VAS] score was used for assessing the generic health-related QoL) through an online survey platform named WenjuanStar (https://www.wjx.cn). A printed questionnaire was provided to those who could not use their mobile phones.

1. Chinese version of the IBD-Control
  The IBD-Control questionnaire is a standardized instrument designed to assess and measure the level of IBD control by examining various aspects of symptoms, treatment effectiveness, and overall QoL. It consists of four sections (13 categorical items) and a VAS. Disease control perception is assessed using an eight-item scale along with a VAS. The eight-item scale (comprising the first and third sections of the 13 items) ranges from 0 to 16, with higher scores indicating a greater sense of disease control. VAS ranges from 0 (worst possible disease control) to 100 (best possible disease control) (S1 Fig in S1 File).
2. Short health scale
  The short health scale (SHS) was developed by Dr. Hjortswang in Sweden in 2006; it is a rapid and specific measurement tool that has been used to assess QoL in patients with IBD in clinical trials and practice [24]. It comprises four items: symptoms, function, worry, and well-being. Each item represents the QoL domain. The items are graded on a 10 cm VAS. Higher VAS scores indicate worse QoL. It has been proven to have good validity and reliability in Chinese patients [25].
3. EQ-5D-5L
  The EQ-5D-5L is a widely used generic health-related quality of life (HRQoL) instrument [26]. It consists of five dimensions: mobility, self-care, usual activities, pain/discomfort, and anxiety/depression. Each dimension has five levels of severity, ranging from no problems to extreme problems, and each dimension has five response levels: no problems, slight problems, moderate problems, severe problems, and extreme problems. It demonstrates good reliability, validity, and responsiveness in a Chinese context.
4. Disease activity measurements
  HBI is a clinical tool utilized for the assessment of disease activity in individuals with CD. The index encompasses a set of parameters including general well-being, abdominal pain, number of liquid stools per day, abdominal mass, and extra intestinal manifestations. Each parameter is assigned a numerical score with higher scores indicating more pronounced disease activity. ≤4 indicates clinical remission. SCCAI is a validated tool utilized to quantify disease activity in patients with UC, composing bowel frequency, rectal bleeding, urgency, stool consistency, and general well-being. The calculated score ranges from 0 to 20, where active disease is a score of 5 or higher. The Physician Global Assessment (PGA) is also utilized to record disease activity, such as mild, moderate, and severe activity, as well as remission. The physicians involved in the evaluation were all IBD specialists who had undergone rigorous training and had extensive clinical experience in managing IBD patients.

#### Psychometric properties

The internal consistency was evaluated by Cronbach’s α with a score greater than 0.70 as an optimal threshold [27]. Test-retest reliability was analyzed by asking a subset of patients to complete the questionnaires a second time after 2 weeks. Patients who reported no alteration in their disease condition based on question 2 of the IBD-Control questionnaire were used to assess test–retest reliability.

A qualitative method was used to assess face validity. Ten hospitalized patients diagnosed with IBD were administered the Chinese version of the IBD Control questionnaire to assess the difficulty, ambiguity, and clarity of the words in each question item.

To evaluate the discriminant ability, we examined whether there were statistically significant differences in IBD-Control scores among patients with different levels of disease activity.

The convergent validity was assessed by calculating the Pearson correlation coefficients between the scores of the IBD-Control-8 and measurements of QOL as well as clinical disease activity. We hypothesized that there would be moderate (>0.30) to strong (>0.50) correlations between scores of IBD-Control-8 and both QoL and clinical disease activity. Additionally, we hypothesized that the correlations between the IBD-Control-8 scores and disease-specific QoL measures (SHS) would be stronger compared to the correlations with generic QoL measures (EQ-5D-5L questionnaire).

To evaluate the structural validity of the Chinese version of the IBD-Control questionnaire, we conducted an exploratory factor analysis (EFA). The adequacy of the sample for EFA was assessed using the Kaiser-Meyer-Olkin (KMO) measure, with a value >0.70 considered acceptable [28]. Bartlett’s test of sphericity was also performed, with a significant p-value (p<0.05) indicating the data was suitable for factor analysis [29].

Receiver operating characteristics (ROC) analysis was used to assess the capability of the IBD-Control questionnaire in distinguishing patients with good disease control. A state of "quiescent" disease was defined by considering the following parameters: HBI score less than 4 for CD or a SCCAI score less than 4 for UC, PGA of remission without any escalation of IBD medication, not awaiting surgical treatment, and no reported worsening of gastrointestinal symptoms in the past two weeks. Our analysis led to the identification of an optimal cut-off score for IBD-Control-8, which should ideally demonstrate a high specificity exceeding 80%. The discriminative accuracy was assessed by calculating the area under the curve (AUC), with an AUC value greater than 0.7 determined as acceptable [30].

### Data analysis

Means and SD were presented for normally distributed continuous outcomes, whereas medians with interquartile ranges (IQRs) were presented for non-normally distributed outcomes. Categorical variables were expressed as percentages (%). Statistical significance was defined as a p-value less than 0.05. All statistical analyses were conducted using SPSS software (version 25.0; IBM Statistics, Armonk, NY, USA).

#### Ethical considerations

This study was conducted in accordance with the Declaration of Helsinki and approved by the Shandong University Qilu Hospital Medical Ethics Committee (number: KYLL-202204-005) in April 2022. All participants provided written informed consent.

## Results

### Translation and cultural adaption

The Chinese version of the IBD-Control was finalized after forward translation, backward translation, a pilot test, and an expert panel review. Minor adjustments were made to account for cultural background, linguistic expression, and disease-specific considerations. Notably, the S-CVI/Ave was 0.98, thereby suggesting a high level of content validity.

### Patient characteristics

In total, 155 patients were included in the present study. A total of 150 patients completed the questionnaire, with a completion rate of 96.8% (150/155). Patient characteristics are presented in Table 1. A total of 119patients (79.3%) had UC and 31 (20.7%) had CD. Among these, 92 were male and the mean age was 40.94±16.3 years.

**Table 1:** Demographic and clinical factors of patients with IBD.

Variable	Level	CD (n = 31)	UC (n = 119)
Age (years, mean±SD)		30.49±11.51	43.73±13.32
Disease duration (years, M[Q25, Q75])		1(0.5,2.5)	5(2.1,9.8)
Gender (n (%)	male	22 (70.9)	70(58.8)
	female	9 (29.1)	49 (41.2)
Marital status (%)	unmarried	15 (48.4)	24 (20.2)
	married	16 (51.6)	95(79.8)
History of operation (%)	yes	7 (22.6)	3 (2.5)
	no	24 (77.4)	116 (97.5)
Extraintestinal manifestations (%)	yes	12 (38.7)	23 (19.3)
	no	19 (61.3)	96 (80.7)
Medication (%)	5-aminosalicylic acid	10 (32.3)	85(71.4)
	Corticosteroid	7 (22.6)	45 (37.8)
	Immunosuppressants	1 (3.2)	2 (1.6)
	Biologic agent	17 (54.8)	50 (42.0)

SD: Standard deviation; IBD: Inflammtory bowel disease.

### Face validity

The findings of the qualitative face validity of the scale revealed that all 10 patients demonstrated comprehension of the scale’s content and evaluation criteria. Moreover, they expressed consensus regarding the scale’s comprehensiveness and accessibility, perceiving it as straightforward and easily understandable.

### Internal consistency

The Cronbach’s alpha of the IBD-Control-8 was 0.827 for CD and 0.819 for UC, which indicates good internal consistency for both CD and UC populations. For CD patients, the correlations between individual items and total scores ranged from 0.485 to 0.892, while for UC patients, they ranged from 0.558 to 0.712, thereby indicating a moderate to strong relationship between each item and the overall total score (Table 2).

**Table 2:** Item-total correlation of each individual question in IBD-Control-8.

Questions		Item-total correlation	
		CD	UC
Q1a	Do you believe that your IBD has been well controlled in the past 2 weeks?	0.585^a^	0.671^b^
Q1b	Do you believe that your current treatment is useful in controlling your IBD?	0.443^a^	0.614^b^
	In the past 2 weeks, did you:		
Q3a	… miss any planned activities because of IBD	0.727^b^	0.702^a^
Q3b	… wake up at night because of symptoms of IBD	0.596^a^	0.619^b^
Q3c	… suffer from significant pain or discomfort	0.837^b^	0.664^b^
Q3d	. . . often feel lacking in energy (fatigued)	0.891^a^	0.649^a^
Q3e	… feel anxious or depressed because of your IBD	0.687^b^	0.625^b^
Q3f	… think you need a change to your treatment	0.596^a^	0.686^b^

a: p<0.05, b: p<0.001.

### Test-retest reliability

Thirty nine participants were enrolled in the test–retest reliability test. Of the 39 patients, 30 participants indicated no change in disease control during the preceding two-week period, as assessed by the Q2 in IBD-Control. Of the 30 participants, no statistically significant differences were observed in either the IBD-Control-8 subscores or the VAS scores. The mean scores were 75.23±17.33 versus 72.10±14.99 for VAS scores (*r* = 0.894, *p<*0.01) and 12.75±3.49 versus 12.80±3.29 for IBD-Control-8 subscores (*r* = 0.963, *p<*0.01), respectively.

### Convergent validity

The findings revealed noteworthy moderate-to-strong associations among IBD-Control, disease activity, and QoL. Consistent with our hypothesis, we found more robust correlations between IBD control and disease-specific QoL (SHS) compared to generic health-related QoL (EQ-5D-5L) (Table 3).

**Table 3:** Convergent validity.

Measures	Overall		CD		UC	
	IBD-Control-8	IBD-Control-VAS	IBD-Control-8	IBD-Control VAS	IBD-Control-8	IBD-Control VAS
HBI	-	-	-0.356*	-0.275*	-	-
SCCAI	-	-	-	-	-0.654*	-0.590*
PGA	-0.655**	-0.523**	-0.209**	-0.412	-0.576**	-0.497**
SHS	0.675*	0.671**	-0.731	-0.706	-0.489*	-0.554*
EQ-5D-5L	0.555*	0.503*	0.690*	0.652	0.379*	0.455*

IBD: inflammatory bowel disease; VAS: visual analogue scale; HBI: Harvey-Bradshaw index; SCCAI: Short Clinical Colitis Activity Index; SHS: Short health scale; PGA: Physician Global Assessment.

### Structural validity

The exploratory factor analysis (EFA) results demonstrated adequate sample adequacy, with a Kaiser-Meyer-Olkin (KMO) measure of 0.823 and a significant Bartlett’s test of sphericity (χ2 = 342.325, p<0.001). Principal component analysis with varimax rotation extracted 2 factors with eigenvalues greater than 1, accounting for 62.752% of the total variance. The exploratory factor analysis revealed a two-factor structure for the 8-item Chinese version of the IBD-Control questionnaire. However, considering the theoretical model underlying the original scale and the content of the individual items, the research team, after consulting the original authors, unanimously agreed that the two extracted factors were not suitable to be treated as distinct dimensions. Instead, the decision was made to adhere to the single-dimensional structure of the original IBD-Control scale.

### Discriminant ability

**Fig 1:**
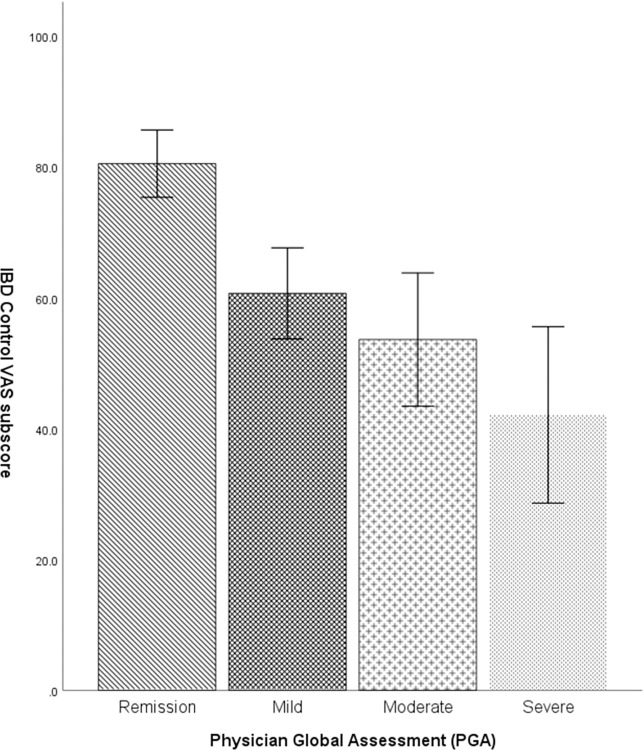


**Fig 2:**
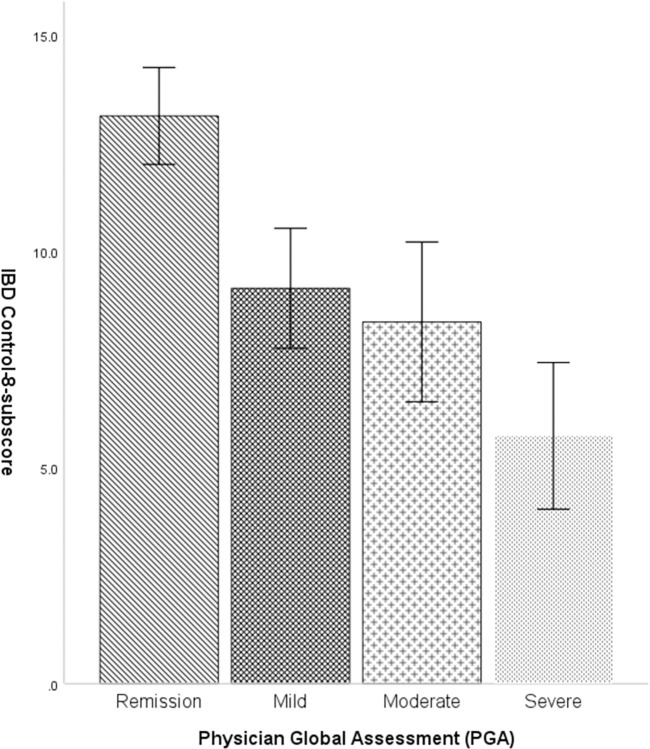


As expected, there were significant differences in the IBD-Control-8 subscores and VAS scores among different levels of disease activity based on the PGA. The mean IBD-Control-8 subscores for patients with different levels of disease activity were as follows: 13.8 (±3.6) for those in remission, 9.35 (±4.2) for those with mild activity, 8.57 (±3.93) for those with moderate activity, and 5.66 (±4.35) for those with severe activity. Mean VAS scores were respectively 81.3 (±17.5), 61.0 (±21.8), 54.2 (±20.1) and 43.7 (±34.9) (Figs 1 and 2).

### Identifying quiescent patients

**Fig 3:**
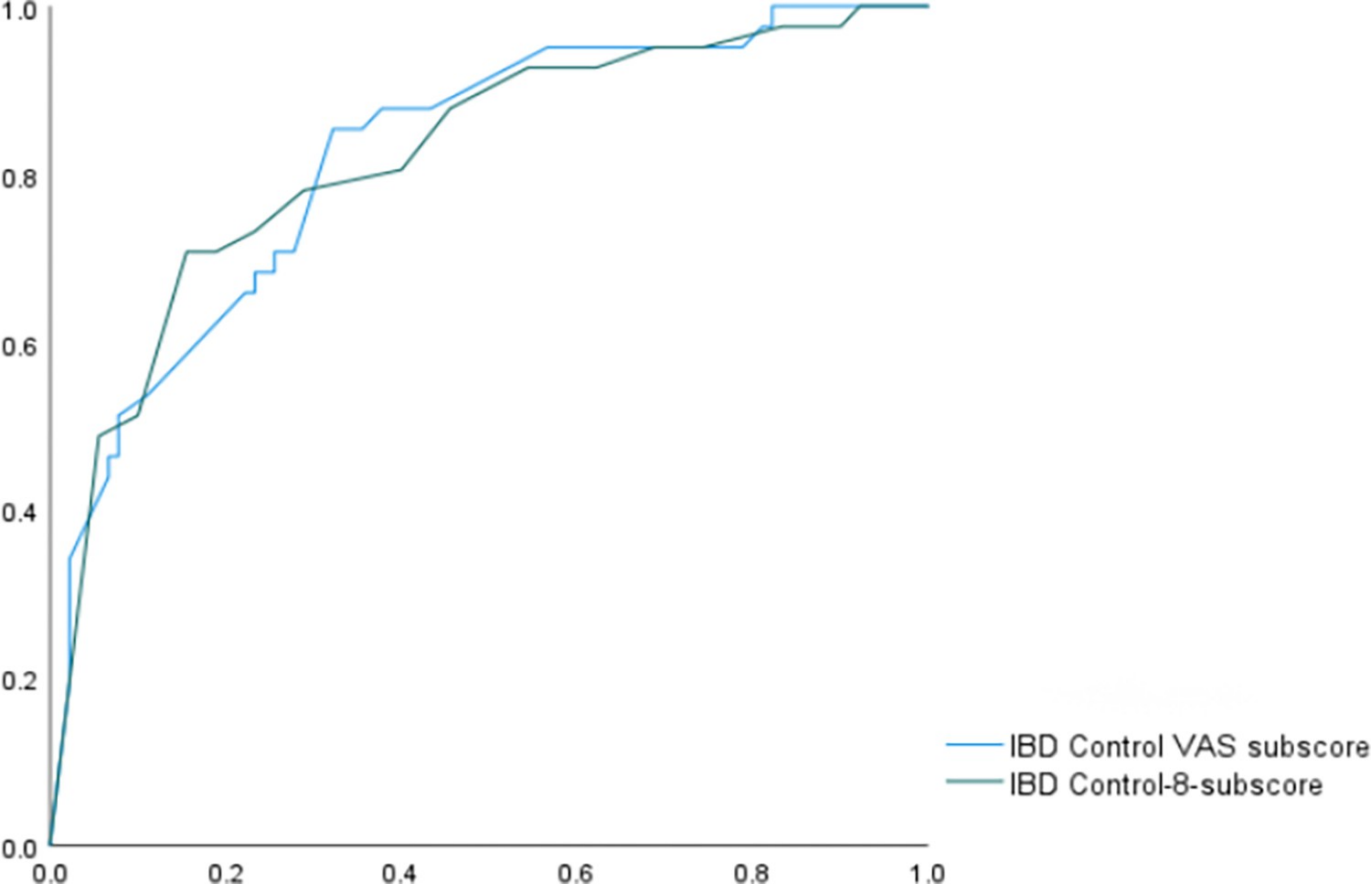


According to our prespecified criteria, 32 of 119 (26.9%) UC participants and 15 of 31 (48.4%) CD participants could be categorized as ‘quiescent’. The AUC was 0.83 (Fig 3). The ROC analysis indicated that the optimal cut-off point for the IBD-Control-8 subscore was 14 points, resulting in a sensitivity of 70.9% and a specificity of 83.5%. Additionally, the cut-off for the IBD-Control VAS score was 70, yielding a sensitivity of 84.4% and a specificity of 69.5%.

## Discussion

Our study involves the first formal translation of an IBD-Control questionnaire from English to Chinese. Additionally, our study focuses on assessing the psychometric properties of the translated IBD-Control questionnaire. Our results shows that the Chinese version of the IBD-Control questionnaire exhibits acceptable psychometric properties, thereby establishing its suitability for clinical utilization in China.

The use of the PROMS in clinical application is being progressively promoted as a means to facilitate patient-centered care, providing valuable information for decision making, and enhancing the quality of healthcare services [31]. Although different PROMs for patients with IBD have been developed, translated, and validated, none are widely used in daily clinical context because of inadequate capture of patients’ disease control perspectives and the length of the multi-domain questionnaires. Previous studies have shown that shortened forms possess higher viability for clinical implementation [32]. In comparison to previous PROMS for IBD, it possesses two major advantages: First, the validated Chinese version of IBD-Control is feasible for use and requires no training in patients. Second, the development of the IBD-Control questionnaire involved close collaboration with patients, ensuring that it captures their personal perception of disease control, in contrast to many other PROMs in IBD that primarily focus on quantifying the extent or intensity of symptoms or concerns. The findings of the present study indicate that IBD-Control can serve as a PROM for triaging IBD patients in China and can complement routine care. The IBD Control consists of 13 categorical items and a VAS, and most participants in our study completed the translated version of the IBD-Control within 2 minutes. These results are consistent with those reported in other countries [11,13,15]. Accordingly, IBD-Control is a user-friendly measurement for patients with IBD to use to determine their perspectives on disease control.

Despite its simplicity, the Chinese version of the IBD-Control scale has proven to be reliable and valid. A full range of psychometric properties was evaluated to conform to current international standards [33–35]. The construct validity was evaluated by comparing the IBD-Control scores with QoL and disease activity. The results showed that good disease control was associated with higher QoL scores and lower disease activity indices (SCCAI/HBI and PGA) in accordance with the expected outcomes. In patients with IBD, an increase in disease activity often correlates with heightened severity of symptoms reported by patients. Consequently, as disease activity escalates, patients may report increased levels of pain, diarrhea, fatigue, among other symptoms, which would be reflected in their IBD-Control scores. Therefore, the disease activity could be used to test the discriminant ability and construct validity. Additionally, the IBD-Control can be used to reciprocally reflect disease activity. Moreover, as anticipated, we discovered a robust correlation between self-reported disease control and disease-specific QoL (measured by SHS) in comparison to generic QoL (assessed by EQ-5D-5L), in accordance with results of previous research elsewhere [13].

The cut-off points of the Chinese version of the IBD-Control-8 were 14 and 70 for IBD-Control-VAS, compared to 13 for IBD-Control-8 and 85 for IBD-Control-VAS in the original version. There are two underlying factors contributing to this discrepancy. First, in our study, the participants consisted of inpatients, of whom 34% were in a phase of disease remission. In contrast, the original study included outpatients, 44% of whom were in remission. Hospitalized patients are more likely to be in the active phase of their illness or require a change in the treatment approach. Consequently, they may experience a lower sense of disease control. Moreover, the hospital environment, with its intensive medical interventions and unfamiliar surroundings, can contribute to a sense of vulnerability, diminishing the perceived control that patients have over their illness [31]. Second, there are differences in the language, ethnicity, and culture between Eastern and Western countries. Under the influence of Eastern cultures, patients may exhibit a higher tendency towards self-doubt and lack of confidence during disease control, even in cases where their illnesses may not be as severe [36]. These cultural norms may discourage individuals from expressing assertiveness or questioning medical professionals, leading to a greater reliance on HCPs and a perceived lack of control over their health. Third, the sample size was insufficient for such an analysis; however, our study did not aim to establish a specific cut-off point, and to validate the optimal cut-off point, future research with larger sample sizes is necessary. Further deliberation is warranted regarding the use of cut-off value analysis for VAS scores. VAS can accurately reflect the degree of disease control with the full range of 0–100. Employing cut-off values may diminish the efficacy of determining patients’ perception of disease control.

The Chinese version of the IBD-Control questionnaire holds significant promise for enhancing the management of IBD patients in China. First, as a simple and user-friendly tool, it can serve as a screening instrument to quickly assess patients’ perceived disease control status, enabling clinicians to tailor their management strategies to the individual needs of patients at various control stages. Second, the questionnaire results can reflect patients’ satisfaction and adherence to the current treatment regimen, guiding clinicians to modify the therapeutic plan accordingly to better meet the individual’s needs. Third, regular administration of the IBD-Control questionnaire can help monitor the dynamic changes in patients’ disease control, enabling timely identification of issues and optimization of the follow-up management [37]. Moreover, the ability of the questionnaire to identify well-controlled patients can help reduce unnecessary clinic visits, thereby optimizing the allocation of limited healthcare resources and improving the overall efficiency of IBD care [38].

Our study had several limitations. First, the participants were inpatients from a single hospital, resulting in an inherent potential for selection bias. The generalizability of these findings to outpatient populations and patients from other geographical areas remains unclear. However, given that the translated versions of IBD-Control in other countries have been validated among outpatients, it is reasonable to assume that the Chinese version will also prove valuable for use in similar patient populations. Second, the sample size was small when compared with other research [13,15]. The prevalence rate demonstrates a relatively lower prevalence compared to Western nations. Nevertheless, even within the limited simple size, the IBD-Control questionnaire demonstrated satisfactory performance in terms of understandability, internal consistency, test-retest reliability, inter-rater reliability, content validity, and discriminant ability.

This study marks the inaugural formal translation of the IBD-Control questionnaire from English to Chinese. Furthermore, we employed an electronic format of the IBD-Control questionnaire, diverging from the original study’s utilization of a paper-based version. Nevertheless, there exists a general consensus within the field that patient-reported outcome measures (PROMs) administered via paper and electronic devices yield comparable quantitative results [39]. By virtue of being the first translation and assessing the psychometric properties of Chinese version of IBD control questionnaire, our study lays the foundation for future investigations and clinical applications in the PROMS of IBD research within China. Our study not only expands the scope of available assessment tools but also holds promise for enhancing the quality of care and disease management of IBD patients.

## Conclusion

The Chinese version of the IBD-Control is a user-friendly PROMS with good reliability and validity to capture patients’ viewpoints regarding disease control. Furthermore, a cut-off score of 14 for the IBD-Control-8 subscore presents the potential for efficient identification of patients with disease in a state of remission, which makes it a valuable instrument for rapidly distinguishing IBD patients with well-controlled disease in the context of routine IBD care in China.

## Supporting information

S1 File The original version of the IBD-Control. (DOCX)

## References

[1] Ng SC, Shi HY, Hamidi N, Underwood FE, Tang W, Benchimol EI, et al. Worldwide incidence and prevalence of inflammatory bowel disease in the 21st century: a systematic review of population-based studies. Lancet Lond Engl. 2017;390(10114):2769–78. doi: 10.1016/S0140-6736(17)32448-0 29050646

[2] Kaplan GG, Windsor JW. The four epidemiological stages in the global evolution of inflammatory bowel disease. Nat Rev Gastroenterol Hepatol. 2021;18(1):56–66. doi: 10.1038/s41575-020-00360-x 33033392 PMC7542092

[3] Qiao Y, Ran Z. Potential influential factors on incidence and prevalence of inflammatory bowel disease in mainland China. J Gastroenterol Hepatol. 2020;4(1):11–5. 10.1002/jgh3.12238 PMC7008158 32055691

[4] Cai Z, Wang S, Li J. Treatment of Inflammatory Bowel Disease: A Comprehensive Review. Front Med. 2021;8:765474. doi: 10.3389/fmed.2021.765474 34988090 PMC8720971

[5] Muzammil MA, Fariha F, Patel T, Sohail R, Kumar M, Khan E, et al. Advancements in Inflammatory Bowel Disease: A Narrative Review of Diagnostics, Management, Epidemiology, Prevalence, Patient Outcomes, Quality of Life, and Clinical Presentation. Cureus. 2023;15(6):e41120. doi: 10.7759/cureus.41120 37519622 PMC10382792

[6] Pittet VEH, Maillard MH, Simonson T, Fournier N, Rogler G, Michetti P, et al. Differences in Outcomes Reported by Patients With Inflammatory Bowel Diseases vs Their Health Care Professionals. Clin Gastroenterol Hepatol Off Clin Pract J Am Gastroenterol Assoc. 2019;17(10):2050–2059.e1. 10.1016/j.cgh.2018.11.029 30471455

[7] Marshall S, Haywood K, Fitzpatrick R. Impact of patient-reported outcome measures on routine practice: a structured review. J Eval Clin Pract. 2006;12(5):559–68. doi: 10.1111/j.1365-2753.2006.00650.x 16987118

[8] Nguyen NH, Zhang X, Long MD, Sandborn WJ, Kappelman MD, Singh S. Patient-Reported Outcomes and Risk of Hospitalization and Readmission in Patients with Inflammatory Bowel Diseases. Dig Dis Sci. 2022;67(6):2039–48. doi: 10.1007/s10620-021-07082-3 34110539 PMC8986995

[9] Farren J. Utilizing Patient-Reported Outcome Measures in the Management of Inflammatory Bowel Disease. Yale Sch Med Physician Assoc Program Theses [Internet]. 2020; Available from: https://elischolar.library.yale.edu/ysmpa_theses/10

[10] de Jong MJ, Huibregtse R, Masclee AAM, Jonkers DMAE, Pierik MJ. Patient-Reported Outcome Measures for Use in Clinical Trials and Clinical Practice in Inflammatory Bowel Diseases: A Systematic Review. Clin Gastroenterol Hepatol. 2018;16(5):648–663.e3. 29074448 10.1016/j.cgh.2017.10.019

[11] Bodger K, Ormerod C, Shackcloth D, Harrison M, IBD Control Collaborative. Development and validation of a rapid, generic measure of disease control from the patient’s perspective: the IBD-control questionnaire. Gut. 2014;63(7):1092–102. 24107590 10.1136/gutjnl-2013-305600 PMC4078750

[12] Wong D, Matini L, Kormilitzin A, Kantschuster R, Simadibrata DM, Lyden S, et al. Patient-reported Outcomes: the ICHOM Standard Set for Inflammatory Bowel Disease in Real-life Practice Helps Quantify Deficits in Current Care. J Crohns Colitis. 2022;16(12):1874–81. doi: 10.1093/ecco-jcc/jjac099 35868223 PMC9721458

[13] Ribeiro I., Fernandes C., Silva J., Ponte A. IBD-Control Questionnaire: validation and evaluation in clinical practice. J Crohns Colitis. 2015;9(suppl_1):S264.

[14] Vicente Lidón R, García López S, Corsino Roche P, Boudet Barraca JM, Sanz Segura P, García Cámara P, et al. Translation into Spanish and validation of a short questionnaire to measure the control of inflammatory bowel disease from the patient’s perspective: IBD-Control, EII-Control. Gastroenterol Hepatol. 2022;45(7):524–34. 34428475 10.1016/j.gastrohep.2021.08.001

[15] de Jong ME, Taal E, Thomas PWA, Römkens TEH, Jansen JM, West RL, et al. Cross-cultural translation and validation of the IBD-control questionnaire in The Netherlands: a patient-reported outcome measure in inflammatory bowel disease. Scand J Gastroenterol. 2021;56(2):155–61. doi: 10.1080/00365521.2020.1857430 33300822

[16] Yu Q, Zhu C, Feng S, Xu L, Hu S, Chen H, Chen H, Yao S, Wang X, Chen Y. Economic Burden and Health Care Access for Patients With Inflammatory Bowel Diseases in China: Web-Based Survey Study. J Med Internet Res. 2021 Jan 5;23(1):e20629. doi: 10.2196/20629 33399540 PMC7815453

[17] Zhang Y, Liu J, Han X, Jiang H, Zhang L, Hu J, Shi L, Li J. Long-term trends in the burden of inflammatory bowel disease in China over three decades: A joinpoint regression and age-period-cohort analysis based on GBD 2019. Front Public Health. 2022 Sep 7;10:994619.18: doi: 10.3389/fpubh.2022.994619 36159285 PMC9490087

[18] Williet N, Sandborn W J, Peyrin–Biroulet L. Patient-reported outcomes as primary end points in clinical trials of inflammatory bowel disease[J]. Clinical Gastroenterology and Hepatology, 2014, 12(8): 1246–1256. e6. 24534550 10.1016/j.cgh.2014.02.016

[19] Mokkink LB, de Vet HCW, Prinsen C a. C, Patrick DL, Alonso J, Bouter LM, et al. COSMIN Risk of Bias checklist for systematic reviews of Patient-Reported Outcome Measures. Qual Life Res. 2018;27(5):1171–9. doi: 10.1007/s11136-017-1765-4 29260445 PMC5891552

[20] Kaichun Wu, Jie Liang, Zhihua Ran, Jiaming Qian, Hong Yang, Qianhu Chen, et al. Consensus on diagnosis and management of inflammatory bowel disease (Beijing,2018). Chin J Dig. 2018;38(9):796–813.

[21] Boateng G O, Neilands T B, Frongillo E A, et al. Best practices for developing and validating scales for health, social, and behavioral research: a primer[J]. Frontiers in public health, 2018, 6: 149. 29942800 10.3389/fpubh.2018.00149 PMC6004510

[22] Andre F, Andre C, Emery Y, Forichon J, Descos L, Minaire Y. Assessment of the lactulose-mannitol test in Crohn’s disease. Gut. 1988 Apr;29(4):511–5. doi: 10.1136/gut.29.4.511 3131194 PMC1433525

[23] Walmsley RS, Ayres RC, Pounder RE, Allan RN. A simple clinical colitis activity index. Gut. 1998;43(1):29–32. doi: 10.1136/gut.43.1.29 9771402 PMC1727189

[24] Hjortswang H, Järnerot G, Curman B, Sandberg-Gertzén H, Tysk C, Blomberg B, et al. The Short Health Scale: a valid measure of subjective health in ulcerative colitis. Scand J Gastroenterol. 2006;41(10):1196–203. doi: 10.1080/00365520600610618 16990205

[25] Hou JT, Peng B, Zhang SJ, Luo YX, Chen YM, Cai JZ, et al. The Short Health Scale: A Valid and Reliable Quality-of-Life Scale for Mainland Chinese Patients with Inflammatory Bowel Disease. Palliat Med Rep. 2022;3(1):154–61. doi: 10.1089/pmr.2021.0066 36059905 PMC9438447

[26] Herdman M, Gudex C, Lloyd A, Janssen M, Kind P, Parkin D, et al. Development and preliminary testing of the new five-level version of EQ-5D (EQ-5D-5L). Qual Life Res Int J Qual Life Asp Treat Care Rehabil. 2011;20(10):1727–36. doi: 10.1007/s11136-011-9903-x 21479777 PMC3220807

[27] Cronbach LJ. Coefficient alpha and the internal structure of tests. Psychometrika. 1951;16(3):297–334.

[28] Li N, Huang J, Feng Y. Construction and confirmatory factor analysis of the core cognitive ability index system of ship C2 system operators[J]. Plos one, 2020, 15(8): e0237339. 32833969 10.1371/journal.pone.0237339 PMC7446803

[29] Shrestha N. Factor analysis as a tool for survey analysis[J]. American journal of Applied Mathematics and statistics, 2021, 9(1): 4–11.

[30] Swets JA. Measuring the accuracy of diagnostic systems. Science. 1988;240(4857):1285–93. doi: 10.1126/science.3287615 3287615

[31] Bashir NS, Walters TD, Griffiths AM, Otley A, Critch J, Ungar WJ. A Comparison of Preference-Based, Generic and Disease-Specific Health-Related Quality of Life in Pediatric Inflammatory Bowel Disease. J Can Assoc Gastroenterol. 2023;6(2):73–9. doi: 10.1093/jcag/gwac034 37025514 PMC10071296

[32] Bull C, Byrnes J, Hettiarachchi R, Downes M. A systematic review of the validity and reliability of patient-reported experience measures. Health Serv Res. 2019;54(5):1023–35. doi: 10.1111/1475-6773.13187 31218671 PMC6736915

[33] Frost MH, Reeve BB, Liepa AM, Stauffer JW, Hays RD, Mayo/FDA Patient-Reported Outcomes Consensus Meeting Group; What is sufficient evidence for the reliability and validity of patient-reported outcome measures? Value Health. 2007;10 Suppl 2:S94–105. 17995479 10.1111/j.1524-4733.2007.00272.x

[34] U.S. Department of Health and Human Services FDA Center for Drug Evaluation and Research, U.S. Guidance for industry: patient-reported outcome measures: use in medical product development to support labeling claims: draft guidance. Health Qual Life Outcomes. 2006;4:79. 17034633 10.1186/1477-7525-4-79 PMC1629006

[35] Calvert M, Kyte D, Price G, Valderas JM, Hjollund NH. Maximising the impact of patient reported outcome assessment for patients and society. BMJ. 2019;364:k5267. doi: 10.1136/bmj.k5267 30679170

[36] Ran MS, Hall BJ, Su TT, Prawira B, Breth-Petersen M, Li XH, et al. Stigma of mental illness and cultural factors in Pacific Rim region: a systematic review. BMC Psychiatry. 2021;21(1):8. doi: 10.1186/s12888-020-02991-5 33413195 PMC7789475

[37] Lenti M V, Scribano M L, Biancone L, et al. Personalize, participate, predict, and prevent: 4Ps in inflammatory bowel disease[J]. Frontiers in Medicine, 2023, 10: 1031998. 10.3389/fmed.2023.1031998 PMC10126747 37113615

[38] Ng S C, Mak J W Y, Pal P, et al. Optimising management strategies of inflammatory bowel disease in resource-limited settings in Asia[J]. The Lancet Gastroenterology & Hepatology, 2020, 5(12): 1089–1100. 33181088 10.1016/S2468-1253(20)30298-3

[39] Muehlhausen W, Doll H, Quadri N, Fordham B, O’Donohoe P, Dogar N, Wild DJ. Equivalence of electronic and paper administration of patient-reported outcome measures: a systematic review and meta-analysis of studies conducted between 2007 and 2013. Health Qual Life Outcomes. 2015,13:167. doi: 10.1186/s12955-015-0362-x 26446159 PMC4597451

