# Supplementary figures and images for: Translation, cross-cultural adaptation and validation of the Chinese version of the IBD-Control questionnaire: A patient-reported outcome measure in inflammatory bowel disease
Source: PLoS One. 2024 Dec 12;19(12):e0311529. doi: 10.1371/journal.pone.0311529 (PMC11637382; doi:10.1371/journal.pone.0311529)

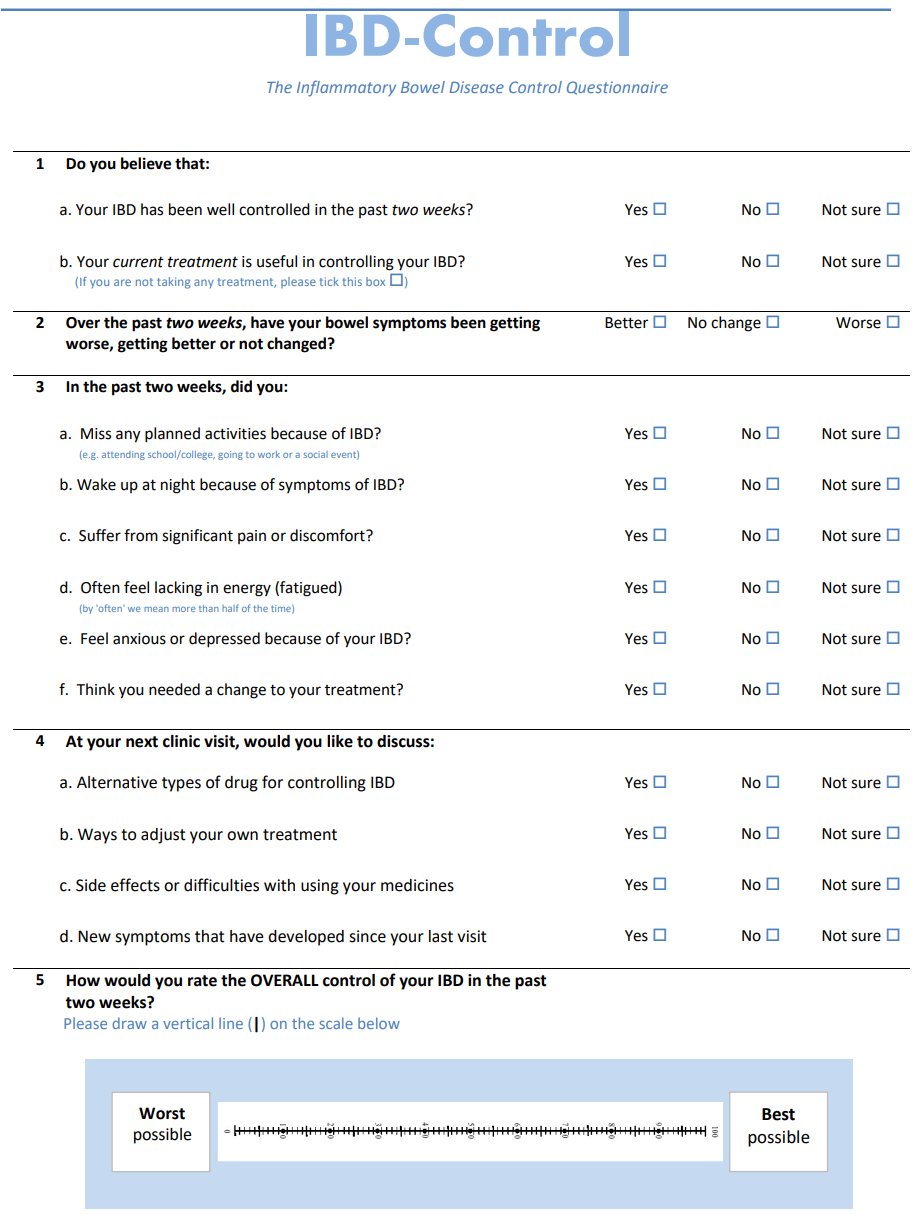

Supplement: S1 File — (DOCX) [file pone.0311529.s001.docx]
